# Online self-management in patients with COPD: with or without the doctor/nurse

**Published:** 2012-06-15

**Authors:** Esther van Noort, T.H Mennema-Vastenhout

**Affiliations:** Curavista BV, The Netherlands; Havenziekenhuis, The Netherlands

**Keywords:** COPD, self management, participation, elderly

## Abstract

**Introduction:**

Curavista Health is an online patient self-management platform. Patients use the platform with or without their doctor/nurse. Fifty centres in Benelux are using the platform for diabetes, hypertension, asthma, COPD, migraine, Parkinson’s Disease, Multiple Sclerosis, breast cancer, prostate cancer, LUTS, bi-polar diseases, chronic pain, Herpes Zoster, GERD, preConsult, and adherence to medical therapy. Many hospitals use one or more programmes. The platform is currently available in Dutch, English and French.

**COPD:**

Chronic Obstructive Pulmonary Disease (COPD) is a chronic disease in the elderly. Airways are increasingly blocked and exacerbations (increase of symptoms) are common. Exacerbations ameliorate the disease and diminish the quality of life. Exacerbations are responsible for most of the augmenting costs of treating COPD. Self-management is a promising method as patients learn to recognize and treat as early as possible exacerbations.

**Case study:**

The case study is called: mijncopdonline.nl. It is a self management programme that consists of 3 basic elements:

- status (CCQ, BMI, FEV1, MRC-dyspnoea, medication)
- self management (how do I feel today and what are my specific actions?)
- eConsult.

Since mid-2008 patients can register online and participate in the programme (without involving their GP/pulmonologist/nurse). The pulmonary division of the Havenziekenhuis (division of the Erasmus MC) started using the programme in October 2010 and incorporated it into their daily routine. All out-patients can participate. The team monitors patients online, exchange eConsultations with patients and discusses the results during the regular visit.

**Results:**

We compared the adherence to the programme in two groups: (1) the group without the doctor/nurse as a coach (n=495) and (2) the group with the doctor/nurse as a coach (n=42). The content of the programme was identical in both groups. Age and gender were similar in both groups and was representative of the overall COPD population of the Havenziekenhuis. In the group without coaching, 65% of participants used the programme once, 22% twice and 13% three times or more. In the group with coaching, 24% used the programme once, 14% twice and 62% three times or more.

**Discussion:**

Self-management is a hot topic. Self-management in itself is not new: patients have been keeping diaries on paper for many years. However, in recent years, online database technology has provided an opportunity to create interactive, personalized, self-management programmes, often referred to as eHealth. The word ‘self-management’ suggests that patients manage themselves and the role of the professional is limited. However, these data suggest that participants make better use of eHealth programmes when supported by healthcare professionals. Therefore eHealth should be embedded into regular care in order to be fully effective.

